# The New Methods of Treatment

**Published:** 1921-06-04

**Authors:** 


### ASTHMA AND HAY FEVER.

### The New Methods of Treatment.

In the latest issue of that valuable publication, the *Medical Annual**, appears an excellent review by Dr. Arthur Latham of the modern tendencies in treating these often baffling conditions. From time to time isolated reports and researches on this or that aspect of the problem have been published, but the practitioner has been left without any comprehensive guide as to how he should set about dealing with a case when met with. In common with other sections of the enormous field covered by the *Annual*, that which we have selected will be found of the greatest use in practice.

### Bronchial Asthma and Vaccines.

In the first place the position is considered with respect to true bronchial asthma. It is quoted that out of a series of eighty-one cases treated with autogenous bacterial vaccines obtained from the flora of the respiratory passages the following results were obtained. These patients were as far as possible selected from those in whom all obvious septic foci had been carefully removed. The majority were typical in that they had suffered over a considerable period and were, by disappointing experience, well versed in the usual "cures." In many instances one or more of the customary nasal or pharyngeal operations had failed to give relief. In 74.6 per cent. there was, after administration of the vaccine, either complete freedom from asthma or a definite decrease in both its severity and frequency. The largest period of complete relief was three years and of relative relief between four and five years. In only 25.4 per cent. was no definite benefit derived from the inoculations, but in none of those cured was a second course of vaccine tried, otherwise the proportion of failures might have to some extent been reduced. In any case, statistics show that success is sufficiently frequent still to justify adoption of the vaccine method.

### Injection of Peptone.

The precise mechanism of the production of asthmatic attacks and the type of stimulus which causes the paroxysms is well illustrated by the therapeutic use of peptone (a parallel to bacterial proteins) as next reviewed. Auld finds that, according to the patient's reaction to peptone, asthma can be divided into two main divisions. The first responds to, and the second resists, peptone treatment. In suggesting the method for any particular case it is well to bear these types in mind. Dr. Latham records that those of the first group present general good health, slight family predisposition, limited duration of the disease, regular recurrence of the attacks, and freedom from actual bronchitis and emphysema. Those of the second include nearly all those with chronic bronchitis and developed emphysema, those showing cyanosis, and any from whom a more or less oppressed condition of the respiration is never absent. Therefore, in a number of asthma cases one cannot hope for any very good results, but, in the first group, peptone of suitable dilution injected on the principle of desensitising the patient to a protein (*e.g.*, antidiphtheritic serum) may be eminently satisfactory. Obviously, there are definite precautions to be taken if the correct effect is to be produced. These are:

"(1) The rate of injection of the peptone and its dilution. (2) The peptone to be used. (3) The indication of the dermal reactions as the case proceeds. If the patient takes peptone well larger doses may succeed, given by very slow injection to avoid reaction. If the patient is sensitive to peptone, however, . . . the larger dose given in this way may precipitate an attack of asthma. Rarely this occurs very quickly—in a few minutes—with concomitant flushing of the face, especially if Witte's peptone be used, which seems to contain a toxic ingredient not present in muscle peptone. The dermal reaction (which may be produced by a Von Pinquet borer) is not of much value at the beginning, as non-asthmatical persons may give it, and it varies considerably in different persons, but as immunisation proceeds it ought to lessen and finally disappear. Experience proves that the immunising injection should never reach the critical point; it only injures the immunity mechanism. As a general rule, also, there ought to be three clear days between the injections."

It appears on the whole that the type of case, *i.e.*, non-bronchitic, which is *not* likely to benefit by a vaccine prepared for catarrhal bacteria, is just that to which peptone treatment can be most frequently applied with success. Therefore, in the two methods, we should have means for dealing with a high proportion of cases. Also clinical differentiation of the types suitable to each is not, in its essentials, difficult.

### Asthma due to Hay Fever.

But there is a further asthmatic type to consider. The condition may arise directly from a sensitiveness to pollen, which state is the true hay-fever. If hay-fever is the root cause of an asthmatic condition, the treatment of the latter is obviously by injection of pollen extract or so-called pollen "vaccine." The treatment of hay-fever asthma, during acute outbreaks, does not differ from that in other forms. The immunising process with either prophylactic or curative aims is conducted on the now well-known lines adopted against hay-fever itself. In practice, after the diagnostic injection of small quantities of some such preparation as "pollacine" has settled the nature and degree of the patient's sensitisation against pollen, then 20 units of the pollen extract are injected subcutaneously, and doses repeated every two or three days with a 15 unit increase up to 150 or 200 units (which is a maximum dose for most adults). When marked catarrhal respiratory symptoms are present at the same time it is both rational and satisfactory to give, alternately with the pollacine,

* Published by John Wright & Son, Ltd., Bristol. Pp. 600. Illustrated. Price 30s. net.

an autogenous bacterial vaccine. Dr. Latham quotes approximately the same figures for such treatment in hay-fever as in catarrhal asthma, forty-five per cent. seasonal cures, forty-three per cent. improvements, and twelve per cent. without benefit. Again we have a proportionate success which justifies the method. As he says, however, when such immunising treatment has been successful (as indicated by a completely negative skin reaction to the irritant protein employed), and yet the asthma persists, then there must be another irritant causing the asthmatic paroxysms and a careful examination must be made to determine a further cause or some unusual protein toxin. Fleming of St. Mary's Hospital has provided a number of useful contributions to this last part of the subject and we must for them refer the reader to this workers own writings.

Protein Sensitisation.

But it is also noteworthy that Sandford at the Mayo Clinic has also done valuable work upon "protein sensitisation," which phenomena lies at the root of these conditions. Out of the 800 people about 200 gave definite skin reactions to proteins. Twenty-eight persons reacted positively to one or other of the animal emanations, the largest number being sensitive to horse dander. About one hundred persons reacted to one or several of the common food proteins, the greatest number being affected by egg-white, others by certain grains, and a few by particular vegetable proteins. Generally speaking, fruit proteins had apparently little effect, but in a few instances bananas was found to be a definite factor in producing the asthma! In connection with bacterial vaccines it is interesting to note that out of 365 tests to staphylococci, white and yellow, there was not a single positive reaction. Of this series of persons all were not necessarily asthmatic, but their percentage susceptibility is very definitely indicated. On the other hand, a number of known catarrhal organisms gave definitely positive effects.

